# Thylakoid biogenesis has joined the new era of bacterial cell biology

**DOI:** 10.3389/fpls.2013.00458

**Published:** 2013-11-13

**Authors:** Jörg Nickelsen, William Zerges

**Affiliations:** ^1^Molecular Plant Sciences, Ludwig-Maximillians-UniversityPlanegg-Martinsried, Germany; ^2^Biology Department, Concordia UniversityMontreal, QC, Canada

**Keywords:** thylakoid membrane, protein complex assembly, cyanobacteria, green alga, photosystem II, biogenesis center

Thylakoid membranes carry out photosynthetic electron transport using some of the most sophisticated macromolecular multisubunit complexes in nature. Recent years have seen major breakthroughs in elucidating the ultrastructure of all core constituents of these complexes, i.e., photosystem II (PSII), the *cytb*_*6*_*f* complex and photosystem I (PS I) (Eberhard et al., 2008; Umena et al., 2011). They are composed of dozens of protein subunits as well as hundreds of organic and inorganic co-factors, most of which are embedded in the lipid bilayer of thylakoid membranes. Despite this profound knowledge on the architecture and function of the thylakoid membrane relatively little is known about how and where these complexes are assembled during thylakoid membrane biogenesis. In general, the biogenesis process includes the highly-ordered, step-wise assembly of proteins, lipids, pigments like chlorophyll (Chl), and carotenoids, quinones, and metal ions which to a large extent is mediated by dedicated assembly factors assisting specific steps (Schöttler et al., 2011; Komenda et al., 2012; Nickelsen and Rengstl, 2013). Thus, fundamental questions discussed here concern the pathways and their cytological organization, by which these proteins as well as their co-factors are synthesized and assembled. How are these processes coordinated in time and space? Recently, analyses of thylakoid membrane biogenesis in both cyanobacteria and green algae have provided the first evidence for specialized membranous compartments which are distinct from functional thylakoid membranes but involved in the synthesis and assembly of at least some photosynthetic components, especially PSII. These findings have occurred amidst an era of modern bacterial cell biology in which advances in the resolution of fluorescence microscopy are being used to reveal an astonishingly high degree of compartmentalization of diverse processes in gram negative bacteria (Montero Llopis et al., 2010; Govindarajan et al., 2012). Here, we summarize our current knowledge and outline open questions on the degrees to which pathways that synthesize and assemble thylakoid membrane complexes and the lipid bilayer occur in specialized biogenic compartments in cyanobacteria and chloroplasts.

**Figure 1:**
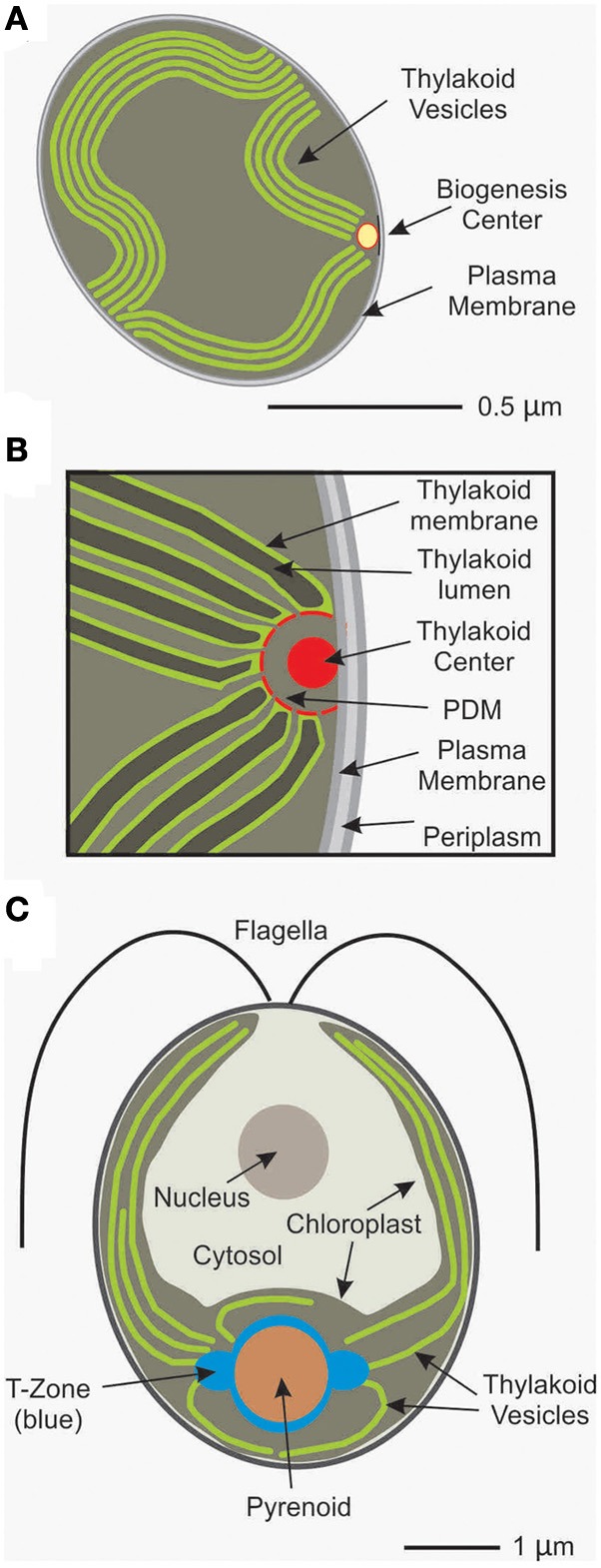
**Illustrations show: (A)** a Synechocystis cell with a biogenesis center and other relevant compartments; **(B)** a higher magnification view of a biogenesis center formed by the central thylakoid center and the surrounding PDMs; and **(C)** a Chlamydomonas cell with the T-zone and other intracellular compartments.

Earlier pioneering work on cyanobacteria detected the core subunits of PSII and PSI apart from thylakoids also in plasma membrane preparations suggesting that the initial phase of the biogenesis process is separated from thylakoids (Zak et al., 2001). Later on, the idea of specialized membranes that are dedicated to the biogenesis of the photosynthetic apparatus was further supported by the identification of the periplasmic PratA factor in *Synechocystis* sp. PCC 6803. PratA represents a tetratricopeptide repeat protein which binds Mn^2+^ ions and delivers them to PSII pre-complexes in an initial phase of its assembly (Stengel et al., 2012). Intriguingly, PratA marks a membrane sub-fraction of intermediate density as compared to the plasma membrane and thylakoid membranes. Immunogold localization studies located these “PratA-defined membranes” (PDM) to distinct regions where the ends of thylakoid vesicles converge at the plasma membrane (Stengel et al., 2012; Figure 1A). Therefore, the role of PratA in PSII biogenesis and its localization to a new type of membrane at the cell's periphery, revealed the existence of a compartment that is specialized in early steps in PSII biogenesis.

The fine structure of this biogenesis center has not yet been completely resolved at a 3-dimensional level but some features are emerging: In their central region, an electron dense cylindrical, rod-like structure (50 nm in diameter and ca. 1 μm in length) with 14 fold symmetry is displayed which previously had been named “thylakoid center” (Kunkel, 1982; Van de Meene et al., 2006; Figure 1B). Recently it has been speculated to consist of homomultimeric Vipp1 protein based on structural similarities shared with assemblies of recombinant Vipp1 (Fuhrmann et al., 2009; Nordhues et al., 2012). Vipp1 has been implicated in thylakoid membrane biogenesis in algal chloroplasts putatively by facilitating lipid insertion into photosynthetic complexes and/or by providing a scaffold defining a biogenic subcompartment (Nordhues et al., 2012). However, the rod-like thylakoid center appears to be partly surrounded by a membranous semicircle probably representing PDMs from which thylakoid membrane lamellae seem to originate (Stengel et al., 2012; Figure 1B). The current working model for these biogenesis centers predicts that PSII assembly, including preloading with Mn ions, is initiated here and further assembly of complexes—assisted by additional assembly factors—then takes place upon the transfer of PSII intermediates into the developing thylakoid lamellae where they finally function in photosynthesis (Figure 1B).

In addition to machineries for protein assembly, metal ion cofactor incorporation, lipid insertion into the membrane bilayer, also Chl synthesis appears to be—at least partially—localized to PDMs as exemplified by the accumulation of the Chl precursor molecules and Chl synthesis enzymes in PDMs (Schottkowski et al., 2009; Rengstl et al., 2011). Therefore, the emerging picture is that the biogenesis center serves as nucleation point for the integration and coordination of the metabolic pathways which are involved in generating functional thylakoid membranes, i.e., protein and Chl synthesis and assembly for PSII biogenesis, lipid insertion to form the bilayer, and transition metal incorporation.

As mentioned above, thylakoid centers have been described in several cyanobacteria suggesting that the concept of compartmentalized biogenesis is more widespread amongst bacteria. Intriguingly, this appears to hold also for the primordial cyanobacterium *Gloeobacter violaceus* which does not contain internal thylakoid membranes but inserts its photosynthetic complexes into its plasma membrane. In *G. violaceus* a clear separation of “orange” membrane fractions accumulating photosystem assembly factors and photosynthetically active “green” patches can be observed (Rexroth et al., 2011). Thus, cyanobacteria appear to have developed an elaborated system of membrane differentiation which ensures the spatial coordination of—at least PSII—assembly steps in biogenic compartments and their separation from active sites of photosynthesis.

In chloroplasts of eukaryotic algae and land plants, how and where the components of thylakoid membrane are synthesized and assembled have been explored for decades (Sato et al., 1999; Zerges, 2000). Chloroplasts originated during evolution some 1.5 billion years ago as a cyanobacterial endosymbiont within the cells of the primordial ancestor of plants and algae. Since then, most of its genes underwent gradual transfer to the nucleus, other genes were lost, and some 100–200 genes were retained in the organelle, with the precise number depending on the species (Green, 2011). Consequently, convergent pathways in these different intracellular compartments supply the polypeptide subunits of the photosynthesis complexes of two convergent protein expression pathways. This situation requires regulatory coordination of the expression and routing of these subunits across different intracellular compartments. Therefore, thylakoid membrane biogenesis in chloroplasts involves a more logistically complex situation than in cyanobacteria. Nevertheless, most of the known steps and biochemical factors in photosystem assembly are conserved in chloroplasts and cyanobacteria (Nickelsen and Rengstl, 2013).

Evidence has also emerged for the localized synthesis and assembly of subunits of PSII in the unicellular green alga *Chlamydomonas reinhardtii*. The single chloroplast in each *C. reinhardtii* cell has a stereotypical cytology (Figure 1C). The localization of translation for chloroplast genome-encoded PSII subunits for the *de novo* assembly of this complex was addressed by *in situ* characterizations of the fluorescence signals from specific mRNAs (encoding subunits of PSII and PSI), chloroplast ribosome subunits, and PSII-specific translation factors by confocal microscopy (Uniacke and Zerges, 2007, 2009; Bohne et al., 2013). Locations of translation were defined as sites where mRNAs and these proteins of the chloroplast translation machinery co-localized when the PSII core subunit synthesis was induced for *de novo* assembly of the complex by light. Based on these criteria, a specific cytological region was detected around the pyrenoid (Figure 1C). In this “translation (T)-zone” the PSII subunit-encoding mRNAs and translation marker proteins co-localized in two to three distinct punctate foci on lateral sides of the pyrenoid and diffusely around the pyrenoid perimeter (Uniacke and Zerges, 2007). The localization of the mRNAs and ribosomes occurred independently of translation, pointing to localization information in the mRNAs and, possibly, the ribosome subunits rather than in the nascent polypeptides (Uniacke and Zerges, 2009). Steps of PSII assembly appear to also occur in the T-zone because unassembled D1 subunits and incompletely assembled PSII complexes were seen to be localized around the pyrenoid (Uniacke and Zerges, 2007). Further support of the T-zone as a specialized location of PSII subunit synthesis and assembly was provided by the identification of membranes with properties expected for such a compartment. This “chloroplast translation membrane” was shown to be (1) none of the known membranes of the chloroplast and (2) have markers of PSII subunit synthesis and assembly (Schottkowski et al., 2012; Bohne et al., 2013). The chloroplast envelope near the T-zone was also seen to be enriched in markers for the machinery that imports thylakoid proteins that are encoded by the nuclear genome and synthesized in the cytosol (Schottkowski et al., 2012). Therefore, PSII subunit synthesis and assembly appears to occur in a biogenic membrane type which is located in the T-zone in *C. reinhardtii*.

Is the T-zone specialized in PSII biogenesis or also a compartment for the synthesis and assembly of other components of the photosynthesis complexes or lipid bilayer of thylakoid membranes? The data for PS I indicate that the T-zone is not where its subunits are synthesized and assembled; a chloroplast mRNA encoding a PSI subunit and a PSI-specific assembly factor (Ycf4) localized neither to the T-zone nor cofractionated with chloroplast translation membranes (Uniacke and Zerges, 2009). It has been suggested that PSI assembly takes place at the eyespot, a photoreceptive structure in the *C. reinhardtii* chloroplast, because Ycf4 was found in the eyespot proteome and in a complex with an eyespot photoreceptor (Kreimer, 2009). The possibility that lipid incorporation into to thylakoid membranes occurs at the ends of thylakoid vesicles and not only in the T-zone was raised by electron microscopy analyses of the effects of Vipp1 deficiency on thylakoid ultrastructure in the Chlamydomonas chloroplast (Nordhues et al., 2012). The *rbcL* mRNA is translated in a localization pattern resembling the T-Zone (Uniacke and Zerges, 2009). Since *rbcL* encodes the Rubisco large subunit, which is localized in the pyrenoid and not to thylakoid membranes, either the T-zone is not involved only in PSII biogenesis protein or the synthesis of *rbcL* translation may be localized coincidentally near the T-zone to target the large subunit to the pyrenoid (Michael et al., 1991). Future work is required to determine the specificity or generality of the T-zone in the various processes underlying thylakoid membrane biogenesis.

The available data—at least for PSII biogenesis—provide an exciting but still developing picture of the spatial organization of thylakoid membrane biogenesis in both cyanobacteria and chloroplasts. Compartmentalization of these pathways could have two major advantages. First, by increasing local concentrations of substrates and intermediates, compartmentalization could favorably shift the chemical equilibrium toward assembly and enable the nearly simultaneous insertion of the different components via substrate channeling. This is apparent in targeted Mn^2+^ loading of PSII at biogenesis centers in cyanobacteria (Stengel et al., 2012). Second, compartmentalization could minimize the release of toxic intermediates from incompletely assembled complexes, such as photoreactive Chl or intermediates in its synthesis. The identification of assembly factors for other complexes will also enable one to test whether PSI and the Cyt*b*_6_*f* complex follow similar routes of biogenesis as does PSII. Finally, it needs to be determined whether or not pathways of thylakoid biogenesis are compartmentalized within the chloroplasts of land plants. In our opinion, chloroplast biology has joined the exciting new field of bacterial cell biology, promising an improved understanding of the cytological organization and molecular mechanisms of thylakoid biogenesis.
